# Automated Volumetric Assessment of Hounsfield Units Using a Deep-Reasoning and Learning Model: Correlations with DXA Metrics

**DOI:** 10.3390/jcm14124373

**Published:** 2025-06-19

**Authors:** Hans K. Nugraha, Vaida Goplin, Linjun Yang, Jonathan M. Morris, Paul M. Huddleston, Mimi C. Sammarco, A. Noelle Larson

**Affiliations:** 1 Department of Orthopedic Surgery, Mayo Clinic, Rochester, MN 55905, USA; nugraha.hans@mayo.edu (H.K.N.);; 2 Orthopedic Surgery Artificial Intelligence Laboratory, Mayo Clinic, Rochester, MN 55905, USA; 3 3D Anatomic Modeling Laboratory, Department of Radiology, Mayo Clinic, Rochester, MN 55905, USA; 4 Limb and Musculoskeletal Regenerative Research Laboratory, Mayo Clinic, Rochester, MN 55905, USA

**Keywords:** bone health, bone mineral density, volumetric Hounsfield unit, artificial intelligence

## Abstract

**Background/Objectives**: Accurate assessment of spinal bone density is essential for evaluating bone health, particularly in preoperative planning. Conventional manual methods for Hounsfield unit (HU) measurements rely on single-slice measurements within the region of interest, limiting their precision and reproducibility in patients with severe vertebral deformities. We hypothesize that a novel deep-reasoning and learning model (DR-AI) can fully automate spinal bone density assessment volumetrically, with high correlations to spinal bone mineral density (BMD) obtained from dual-energy X-ray absorptiometry (DXA), as well as to the T- and Z-scores. **Methods**: A cross-sectional study was conducted on patients who had BMD assessment of their lumbar spine and lumbar CT scans within 1 year. The fully-automated DR model was utilized to analyze the soft-tissue window of the lumbar vertebrae CT scans. Spearman correlation coefficients were calculated to assess the strength of relationships between the computed volumetric HUs and the BMD, T-, and Z-scores from each vertebra. **Results**: 84 patients (67 females, mean age 74.1 ± 10.3 years; 17 males, mean age 68.1 ± 12.4 years) meeting inclusion criteria. Correlation analyses for L1 to L4 showed significant positive relationships (*p* < 0.0001), with the strongest correlation at L2 between HU and BMD (ρ = 0.75). **Conclusions**: the DR model for automated assessment of volumetric HUs offers a highly reliable, efficient, and precise alternative to DXA measurements.

## 1. Introduction

Accurate evaluation of bone density of the spine is essential for assessing general bone quality, especially in preoperative planning for spine surgery. Spinal bone mineral density (BMD) is considered an important factor in surgical planning, including implant selection, screw placement, and postoperative management [1]. Assessment of bone health also plays a critical role in diagnosing conditions such as osteoporosis, which affects millions globally, resulting in increased fracture risk. Osteoporosis is often underdiagnosed due to limited access to screening tools and the silent progression of the disease until a fracture occurs [2]. Effective preoperative assessment of bone density can mitigate risks such as implant failure or subsidence, which are particularly concerning in patients with compromised bone quality [3]. Moreover, understanding bone density distribution within the spine can inform surgical technique, such as use of cement augmentation or opting for medical therapy prior to surgery to improve bone purchase.

Hounsfield units (HUs) are a measure of radiodensity in computed tomography (CT) scans, quantifying X-ray attenuation relative to water (0 HU) and air (−1000 HU), with higher values indicating denser bone (e.g., +300 to +1000 HU). Hus measured on computed tomography (CT) scans offer an alternative for assessing bone density. HU values correlate strongly with biomechanical properties in the thoracolumbar spine and can measure osteoporosis treatment efficacy [4,5]. However, traditional manual HU measurements are labor-intensive, relying on single-slice regions of interest (ROIs) that fail to account for the heterogeneous distribution of bone density within vertebrae [6]. This is particularly problematic in scoliotic patients where osteopenia is more pronounced on the convex side of the deformity [7]. Manual methods are also prone to inter-observer variability and are impractical for routine clinical use. Dual-energy X-ray absorptiometry (DXA) is the gold standard for BMD assessment, providing T-scores and Z-scores to evaluate osteoporosis risk. However, DXA also has limitations, such as false negatives because of osteophytes, soft-tissue calcifications, or obesity, leading to an overestimation of bone mineral content [8]. Furthermore, DXA provides a two-dimensional assessment, which may not fully capture the three-dimensional complexity of vertebral bone structure [9]. This limitation is particularly evident in patients with spinal deformities or degenerative changes, where regional variations in bone density can significantly impact diagnostic accuracy. The reliance on DXA also poses challenges in resource-limited settings, where access to specialized equipment and trained personnel may be restricted, highlighting the need for alternative methods like CT-based HU measurements.

Recent advancements in artificial intelligence (AI), particularly deep learning and reasoning, offer promising solutions for automating bone density assessment. Deep reasoning refers to AI systems capable of complex, multi-step logical analysis, mimicking human-like problem-solving [10]. A novel deep-reasoning and learning-based model, built on top of open-source frameworks for vertebral segmentation [11], has recently been developed to automate volumetric HU assessment. We hypothesize that this model can fully automate volumetric spinal bone density assessment in a broader population, with high correlations to DXA-derived BMD, T-scores, and Z-scores, offering a reliable and efficient alternative for preoperative planning.

## 2. Materials and Methods

Institutional review board approval was obtained prior to initiation of the study (IRB 23-008087).

### 2.1. Study Design

A cross-sectional study was conducted at a single tertiary, academic medical center on all patients who underwent lumbar CT scans from 2014 to 2024. Patient demographic data were retrospectively collected from electronic medical records. Inclusion criteria were as follows: (1) age ≥ 18 years, (2) availability of both lumbar CT scans, and (3) DXA scans of the lumbar spine performed within 1 year. A previous study has demonstrated that for opportunistic HU measurements from CT scans, using a soft-tissue kernel results in negligible errors, as these smoothing kernels are interchangeable without significantly affecting outcomes. However, interchanging with sharpening kernels (e.g., lung, bone, bone plus, or edge) introduced substantial errors, which could critically impact volumetric BMD measurements used for osteoporosis screening and diagnosis [12]. Thus, only soft-tissue kernels were used to minimize such errors and ensure more reliable BMD assessments. Patients with extensive instrumentation, spinal tumor, discitis/osteomyelitis, spinal fracture, and/or vertebroplasty between L1 and L4 were excluded. Contrast-enhanced studies were also excluded, since the HU values in these studies are generally higher on average [13], as well as those obtained intraoperatively, since there were no predictable relationships with values obtained from preoperative CTs [14].

**Figure 1:**
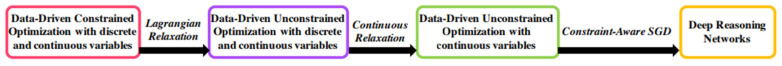
The development flow of the deep-reasoning and learning-based model.

A recently developed deep-reasoning and learning-based model (DR-AI; Deep Reasoning AI Inc., Ithaca, NY, USA) was employed to automate vertebral segmentation and volumetric HU quantification of the cancellous part of the vertebrae, excluding the cortical parts and all possible osteophytes, which may lead to false-negative diagnosis. The model was developed based on publicly available data (deepreasoning3d.com) as well as deidentified patients’ data from our institution. This was performed by an imaging technologist and a research coordinator according to HIPAA guidelines [15]. It integrated deep learning for pattern recognition with reasoning capabilities to solve complex segmentation tasks in an unsupervised or weakly supervised manner [16]. Leveraging an open-source framework for the automated vertebral segmentation [11], it was trained on a dataset of CT scans with annotated vertebral labels (Figure 1). To evaluate the reliability of the automated volumetric HU measurements, random spot checks were performed on a mid-sagittal slice of the CT series using clinical image viewer software (QReads 5.15.3).

The model processed CT scans in the soft-tissue window/kernel to segment each vertebra (L1–L4) and compute volumetric HU values. The segmentation process involved identifying vertebral boundaries and excluding non-bony structures (e.g., cortical bone edges, spinal canal). Volumetric HU was automatically calculated as the mean HU across all voxels within the segmented vertebral body excluding the cortical bone.

### 2.2. Statistics

Descriptive statistics summarized patient demographics and imaging data. Spearman correlation coefficients were calculated to assess relationships between volumetric HU and DXA-derived BMD, T-scores, and Z-scores for each vertebra (L1–L4). The normality of distribution of those values were then assessed using the Shapiro–Wilk test. Assuming non-normality in the distributions, Spearman’s rank correlation coefficient was utilized to assess the correlations between the 4 variables. Correlation strengths were interpreted as weak (r < 0.4), moderate (r = 0.4–0.7), or strong (r > 0.7). Statistical significance was set at *p* < 0.05. Analyses were performed using BlueSky Statistics 10.3.4 (BlueSky Statistics LLC, Chicago, IL, USA).

## 3. Results

**Figure 2:**
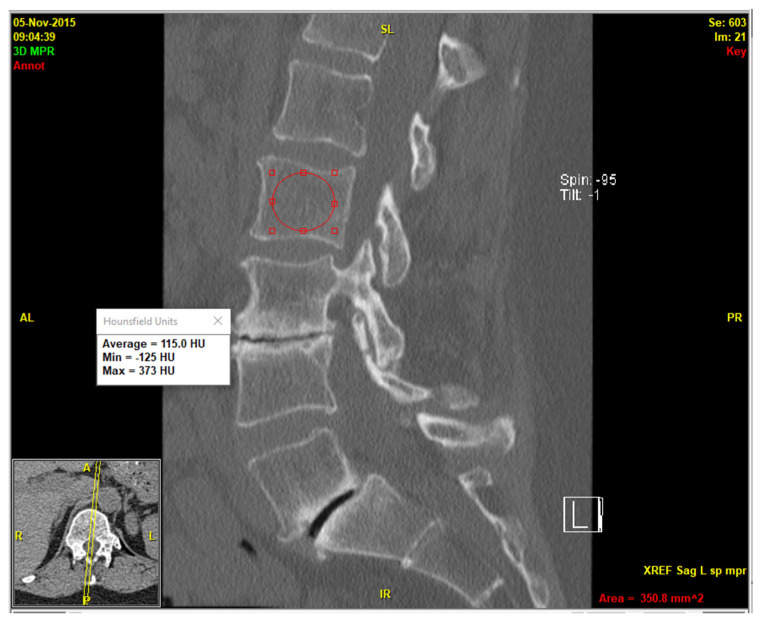
Conventional mid-sagittal method of opportunistic HU measurement.

**Figure 3:**
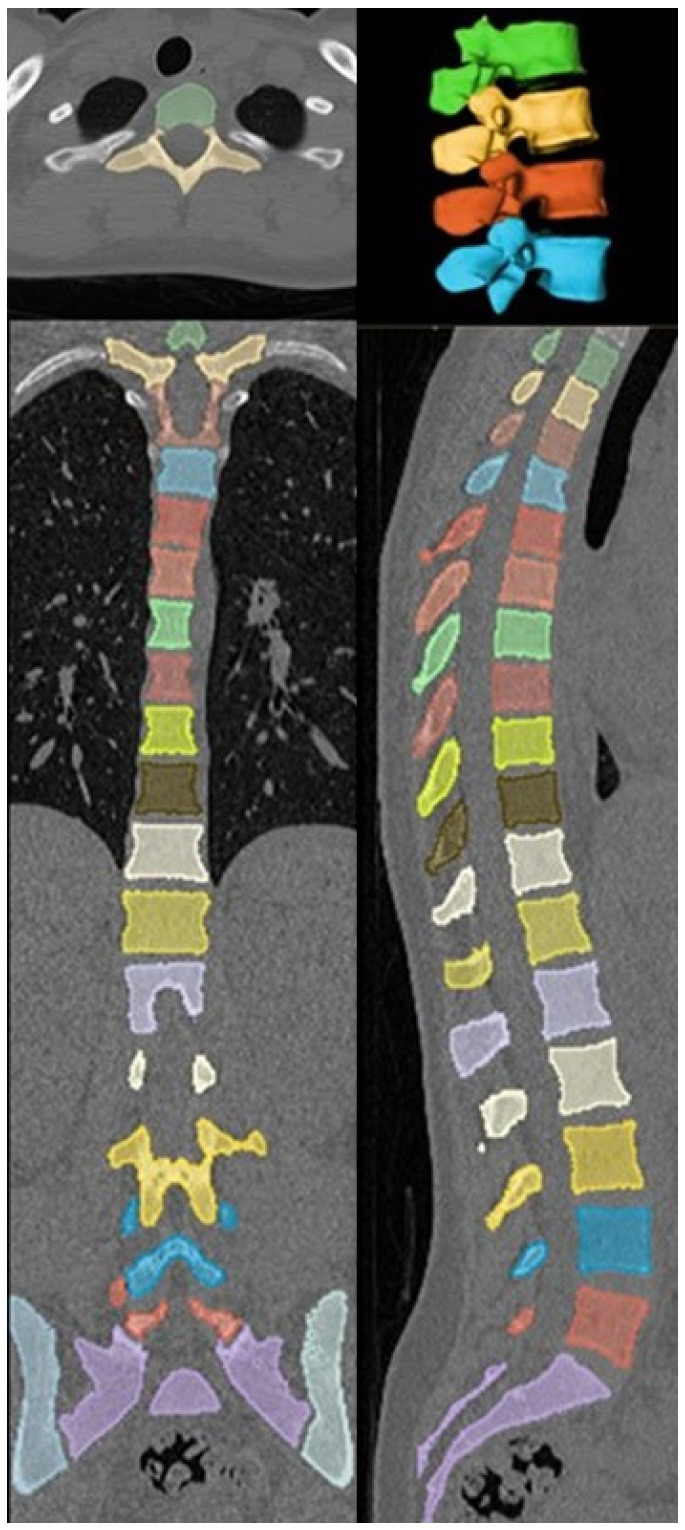
Automated vertebral segmentation generated from the DR-AI model.

A total of 84 patients (67 females and 17 males) met the inclusion and exclusion criteria. All patients were Caucasian, and mean age at imaging was 74.1 ± 10.3 years for females and 68.1 ± 12.4 years for males. The deep-reasoning and learning-based model (DR-AI) successfully demonstrated robust performance in segmenting and quantifying volumetric HU for all L1–L4 vertebrae with high fidelity. Random spot checks confirmed accurate delineation of vertebral boundaries, with no significant discrepancies between automated and manual measurements on mid-sagittal slices (Figure 2). The model processed each CT scan in approximately 30.4 s when hosted on an A100 NVIDIA GPU, (NVIDIA Corp., Santa Clara, CA, USA) providing both the volumetric HU and volume for all scanned vertebrae from all available kernels (Figure 3).

Table 1 shows the average BMD and HU values across all vertebral levels in this study. The Shapiro–Wilk normality test results revealed significant deviations from normality across all vertebral levels and their associated bone health metrics. At the L1 vertebra, the HU value (W = 0.9299, *p* = 0.0002), L1 T-score (W = 0.9353, *p* = 0.0004), and L1 Z-score (W = 0.9458, *p* = 0.0016) all exhibit *p*-values below 0.05, indicating that these measures are not normally distributed. Similarly, the HU-derived BMD at L1 (W = 0.9356, *p* = 0.0004) also rejects the null hypothesis of normality. Moving to L2, the pattern continues with the HU value (W = 0.9493, *p* = 0.0023) and L2 Z-score (W = 0.9502, *p* = 0.0028) showing significant non-normality, as their *p*-values fall below the 0.05 threshold. However, the L2 T-score (W = 0.9662, *p* = 0.0277) and the HU-derived BMD at L2 (W = 0.5667, *p* = 2.8436 × 10^−14^) presented a more extreme case, with the latter having an exceptionally low *p*-value, strongly indicating a non-normal distribution. At the L3 level, the HU value (W = 0.9259, *p* = 0.0001), L3 T-score (W = 0.9422, *p* = 0.0013), and L3 Z-score (W = 0.9645, *p* = 0.0254) all did not follow the normality test, with *p*-values less than 0.05, pointing to non-normal distributions. The HU-derived BMD at L3 (W = 0.9373, *p* = 0.0007) further supported this trend, with a similarly significant *p*-value. The L4 vertebrae, along with their associated metrics, also followed a similar pattern. The HU values at L4 (W = 0.8614, *p* = 2.3554 × 10^−7^) showed extremely low *p*-values, strongly rejecting normality. At L4, the T-score (W = 0.9186, *p* = 0.0001), Z-score (W = 0.9381, *p* = 0.0011), and HU-derived BMD (W = 0.9075, *p* = 3.9826 × 10^−5^) also indicated non-normal distributions, with *p*-values well below 0.05. These findings across all vertebral levels suggested that the bone health data, as measured by HU, T-scores, and Z-scores, consistently deviated from normality; thus, Spearman’s rank correlation coefficient was utilized to assess the correlations between the four variables across all vertebrae from L1 to L4.

The Spearman correlation analyses demonstrated significant positive relationships between Hounsfield unit (HU) values and bone health metrics—bone mineral density (BMD), T-score, and Z-score—across vertebral levels L1 to L4, with all *p*-values less than 0.0001. At the L1 vertebra, Spearman correlation analysis revealed significant positive relationships between the volumetric HU value and bone health metrics, with all *p*-values less than 0.0001. The volumetric HU at L1 showed moderate correlations with BMD (ρ = 0.6633), T-score (ρ = 0.6609), and Z-score (ρ = 0.5168). BMD and T-score were nearly perfectly correlated (ρ = 0.9976), while Z-score strongly correlated with BMD (ρ = 0.8063) and T-score (ρ = 0.8216) (Table 2).

At L2, the correlations between HU and bone health metrics strengthened, with all *p*-values below 0.0001. The volumetric HU value at L2 demonstrated strong correlations with BMD (ρ = 0.7524), T-score (ρ = 0.7549), and Z-score (ρ = 0.5969). The BMD and T-score at L2 were highly correlated (ρ = 0.9389), and Z-score maintained strong associations with BMD (ρ = 0.8628) and T-score (ρ = 0.8249), suggesting that L2 might provide the most robust volumetric HU-based bone health assessment among the vertebrae studied (Table 3).

At L3, the relationships between volumetric HU and conventional bone health metrics slightly weakened, though all *p*-values remain below 0.0001. The volumetric HU value at L3 moderately correlated with BMD (ρ = 0.6130), T-score (ρ = 0.6136), and Z-score (ρ = 0.4410). The BMD and T-score showed a near-perfect correlation (ρ = 0.9972), while Z-score correlated strongly with BMD (ρ = 0.8465) and T-score (ρ = 0.8577) (Table 4).

**Figure 4:**
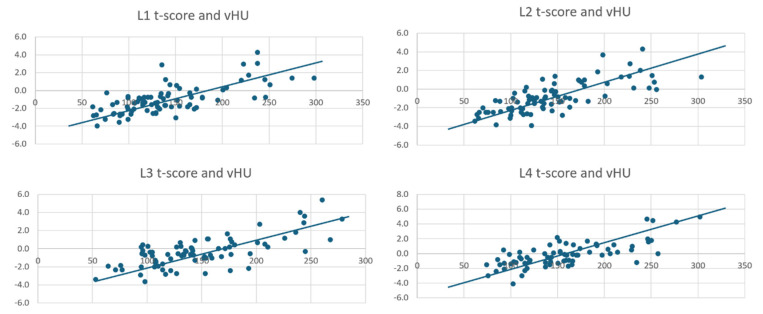
Scatterplots showing data points between the volumetric HU values and T-scores from L1 to L4 vertebrae.

At L4, the volumetric HU value exhibited moderate to strong correlations with bone health metrics, with all *p*-values less than 0.0001. The volumetric HU at L4 strongly correlated with BMD (ρ = 0.6954), T-score (ρ = 0.6942), and moderately with Z-score (ρ = 0.5747). The BMD and T-score are also strongly correlated (ρ = 0.9850), and Z-score showed robust associations with BMD (ρ = 0.8676) and T-score (ρ = 0.8899) (Table 5). All volumetric HU and T-score values can be seen in the scatterplots (Figure 4).

## 4. Discussion

The accurate assessment of spinal bone density is helpful in surgical planning [1]. DXA scans have been the gold standard for BMD evaluation, but have limitations, such as false-negative diagnoses and poor correlation to surgeon intraoperative ratings of bone health [8]. Further, DXA scanning necessitates another study, while Hounsfield units can be used opportunistically for patients undergoing CT scans for other indications. The Bone Health and Osteoporosis Foundation guidelines advise BMD screening for all women aged 65 and older, and men aged 70 and older, regardless of clinical risk factors. Despite this recommendation, a considerable number of older adults still do not undergo central DXA testing [17]. The study’s findings highlight the DR-AI model’s potential to facilitate bone health assessment. Its superior correlations compared to manual methods underscore the importance of volumetric analysis in capturing the complex architecture of vertebral bone. This may be particularly impactful for scoliosis patients, where traditional methods may be prone to sample selection. Exploring its integration with predictive analytics could further enhance its utility, enabling forecasts of surgical outcomes or osteoporosis progression based on longitudinal HU trends.

Opportunistic HUs measured from CT scans have emerged in the past decade as a viable alternative to DXA [18]. They offer strong correlations with biomechanical properties in the thoracolumbar spine and the ability to monitor results of osteoporosis treatment over time [4,5]. By leveraging CT scans performed for other purposes, HUs could serve as a scalable and cost-effective alternative to DXA for many patients, minimizing both extra costs and radiation exposure [19]. The cutoff value for osteoporosis in patients with degenerative lumbar disease is 110 HUs, while for osteopenia, it is 160 HUs [20]. Despite these advantages, however, manual HU measurements are labor-intensive, prone to inter-observer variability, and fail to account for the heterogeneous distribution of bone density within vertebrae, particularly in scoliotic patients where osteopenia is more pronounced on the convex side [6,7]. This study leverages a novel deep-reasoning and learning-based model (DR-AI) to automate volumetric HU assessment, aiming to address these challenges and provide a reliable, efficient alternative for preoperative planning.

Recent advances in AI have been propelled by developments in deep learning, which has achieved remarkable success in areas such as image recognition, face and speech recognition, autonomous driving, and high-fidelity images for video games. Beyond these common applications, AI holds immense potential to significantly accelerate scientific discovery. However, scientific breakthroughs often require a combination of data analysis and reasoning based on prior knowledge, which remains a challenge for AI. In pursuit of more effective AI, researchers have drawn inspiration from human cognition. Kahneman describes human thought as a blend of two systems: System 1, which handles fast, automated pattern recognition tasks, and System 2, which engages in complex reasoning [21]. Both of these cognitive processes have been emulated in AI systems. Deep learning serves as one of the most successful analogs of System 1, with its rapid processing and pattern recognition capabilities, while System 2 is mirrored in AI fields such as combinatorial and constraint reasoning, which involve search and inference to solve complex problems. The key to success in these tasks lies in the interpretable structured latent space, which is essential for incorporating prior knowledge. This latent space is constructed using variables that have clear interpretations and can be incorporated into domain-specific rules. Moreover, prior knowledge often involves complex constraints, such as thermodynamic rules for X-ray diffraction patterns [22]. To encode these discrete variables involved in combinatorial constraints, a group of entropy-based continuous relaxations is projected, gradually minimizing the entropy of the distribution to approximate the original discrete variable [15].

**Figure 5:**
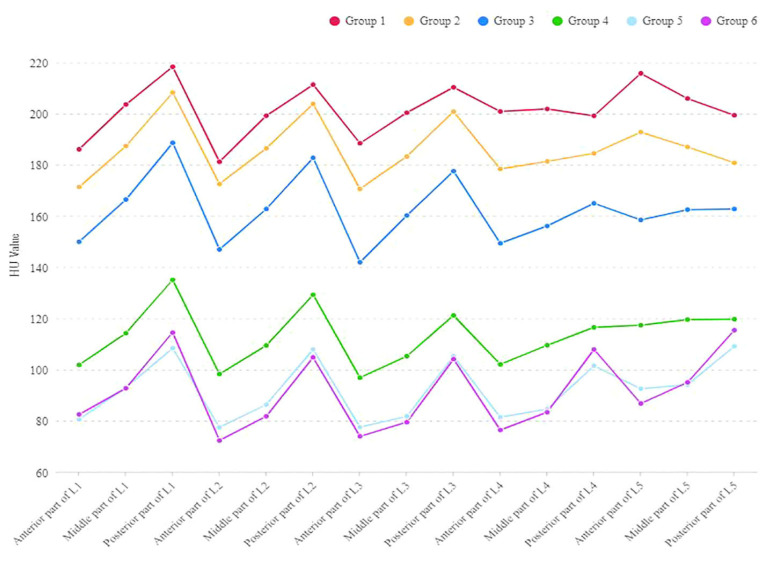
Variability of HU values at the anterior, middle, and posterior parts of L1–L5 vertebral bodies in different age groups [6].

The adoption of a deep-reasoning and learning-based approach offers several advantages over traditional methods. Manual HU measurements, often based on a single slice within the region of interest, fail to capture the full variability of bone density across vertebral levels [8,23]. In contrast, our model leverages volumetric data, providing a more comprehensive and consistent evaluation. This is particularly critical in patients with scoliosis, where bone density may vary due to asymmetric stress stimuli [6,7] (Figure 5). By automating the process, the model also reduces the time and effort required compared to newer, labor-intensive techniques that use VOIs and ROIs across multiple planes [23]. These improvements position the model as a practical and efficient option for clinical use.

The DR-AI model integrates deep learning for pattern recognition with reasoning capabilities to perform complex, multi-step logical analysis, mimicking human-like problem-solving [10,16]. By building on open-source frameworks for vertebral segmentation [11], the model successfully segmented and quantified volumetric HU values for L1 to L4 vertebrae in a cohort of 84 patients, demonstrating robust performance with high fidelity. Random spot checks on mid-sagittal slices confirmed the accuracy of the automated vertebral boundary delineation, with no significant discrepancies compared to manual measurements. The model’s processing time of less than 1 min per CT scan highlights its efficiency, making it a practical tool for routine clinical use compared to the labor-intensive manual methods. This automation addresses a key limitation of traditional HU measurements, which are impractical for widespread adoption due to their reliance on single-slice regions of interest (ROIs) and susceptibility to inter-observer variability [6].

The Shapiro–Wilk normality test confirmed significant deviations from normality across all vertebral levels (L1–L4) for HU, BMD, T-scores, and Z-scores, with *p*-values consistently below 0.05 (e.g., L1 HU: W = 0.9299, *p* = 0.0002; L4 HU: W = 0.8614, *p* = 2.3554 × 10^−7^). This non-normality necessitated the use of Spearman’s rank correlation coefficient, a non-parametric measure, to assess the relationships between these variables, ensuring robust statistical analysis. This finding aligns with previous study, which showed that bone density values for the cervical and lumbar spine, along with most other data, did not follow a normal distribution [24].

The Spearman correlation analyses revealed significant positive relationships between volumetric HU and DXA-derived bone health metrics across all vertebral levels, with all *p*-values less than 0.0001. At L1, volumetric HU showed moderate correlations with BMD (ρ = 0.6633), T-score (ρ = 0.6609), and Z-score (ρ = 0.5168), while BMD and T-score were nearly perfectly correlated (ρ = 0.9976), and Z-score strongly correlated with both BMD (ρ = 0.8063) and T-score (ρ = 0.8216). At L2, the correlations between volumetric HU and bone health metrics strengthened, with HU showing strong correlations with BMD (ρ = 0.7524) and T-score (ρ = 0.7549), and a moderate correlation with Z-score (ρ = 0.5969). The BMD and T-score correlation remained high (ρ = 0.9389), and Z-score showed strong associations with BMD (ρ = 0.8628) and T-score (ρ = 0.8249). At L3, the relationships between volumetric HU and bone health metrics weakened slightly, with moderate correlations to BMD (ρ = 0.6130), T-score (ρ = 0.6136), and a weaker correlation with Z-score (ρ = 0.4410). The BMD and T-score maintained a near-perfect correlation (ρ = 0.9972), and Z-score showed strong correlations with BMD (ρ = 0.8465) and T-score (ρ = 0.8577). At L4, volumetric HU exhibited moderate to strong correlations with BMD (ρ = 0.6954), T-score (ρ = 0.6942), and a moderate correlation with Z-score (ρ = 0.5747). The BMD and T-score correlation was strong (ρ = 0.9850), and Z-score showed robust associations with BMD (ρ = 0.8676) and T-score (ρ = 0.8899).

The variability in correlation strengths between volumetric HU and DXA-derived metrics across L1 to L4 warrants further exploration. At L2, the strongest correlations were observed (e.g., HU vs. BMD: ρ = 0.7524; HU vs. T-score: ρ = 0.7549), potentially due to its central position in the lumbar spine, where bone density may be less affected by degenerative changes or biomechanical stressors compared to L3 or L4. Anatomically, L2 experiences more uniform load distribution, which may result in a more consistent trabecular bone structure [25,26]. In contrast, L3 exhibited weaker correlations (e.g., HU vs. Z-score: ρ = 0.4410), possibly reflecting early degenerative changes or increased heterogeneity in bone density due to its proximity to the lower lumbar region, where spinal curvature and load-bearing demands shift. L1 and L4 showed moderate to strong correlations, suggesting that transitional zones between thoracic and lumbar regions (L1) or increased cortical bone involvement (L4) may influence HU measurements differently.

These differences align with biomechanical studies indicating that vertebral levels experience distinct stress patterns [27]. For instance, the convex side of scoliotic spines bears greater mechanical stress, leading to asymmetric bone density distribution. [7] The DR-AI model’s volumetric approach mitigates this issue by averaging HU values across the entire vertebral body, excluding cortical bone, offering a more representative assessment than single-slice ROI methods. Compared to prior research reporting a correlation of 0.55 between DXA T-scores and single-slice HUs [28], our volumetric HU correlations (ranging from 0.61 to 0.75 with T-scores) demonstrate a marked improvement, underscoring the advantage of capturing three-dimensional bone density heterogeneity.

The DR-AI model’s automation and efficiency (processing times of 0.51 min per CT scan) position it as a transformative tool for clinical practice. In preoperative planning, volumetric HU data can enhance surgical decision-making by providing a comprehensive bone density profile, potentially reducing complications such as screw loosening or vertebral fractures. For example, surgeons could use HU thresholds (e.g., <110 HU for osteoporosis) to tailor implant choices or adjust screw trajectories (using cortical trajectory screw fixation instead of traditional pedicle screw trajectories, or even using AI-based trajectory [29,30]). Those with osteopenic bones would also benefit from having more levels fused, with cement-augmented pedicle screws or bicortical screw purchase. This could be particularly beneficial in minimally invasive spine surgery, where accurate preoperative assessment is essential due to limited intraoperative visibility.

Beyond surgery, the model also holds promise for osteoporosis screening. Given DXA’s limitations in certain populations (e.g., obese patients or those with degenerative changes), volumetric HU could serve as an opportunistic screening tool using existing CT scans, aligning with guidelines from the Bone Health and Osteoporosis Foundation [2] and American Association of Clinical Endocrinologists/American College of Endocrinology [31]. This approach could increase screening rates among at-risk individuals who do not undergo DXA, particularly in settings where CT imaging is already part of routine care. Additionally, the model could facilitate measurement in HU values over time, specifically in patients undergoing concomitant spine treatment or oncology surveillance who also need periodic assessment of bone health [5]. This could be particularly useful in managing patients with atypical fracture risks or those who exhibit poor response to standard therapies, enabling personalized treatment adjustments based on objective data.

Despite its strengths, the study has limitations. The final trained model is not yet publicly available. The sample size (n = 84) is modest, and the cohort’s demographic homogeneity (predominantly older Caucasian females) from a single-center retrospective dataset might limit generalizability. Validation in larger, more diverse populations—spanning different demographics and pathologies—is essential to confirm the model’s robustness. Further validation in cohorts with contrast-enhanced CT scans and hardware to assess might also be needed to explore its versatility. Technical challenges also exist, including the need for standardized CT protocols to ensure HU accuracy across institutions. Integrating DR-AI into clinical workflows requires overcoming barriers such as personnel training. Clinical implementation would also require addressing practical considerations, such as developing appropriate user interfaces for clinicians. After future validation, prospective studies could establish HU-based thresholds for surgical and therapeutic decision-making. The broader implications of this technology extend beyond spine surgery and osteoporosis screening. For instance, the DR-AI model could be adapted for other skeletal regions, such as the hip or femur, where bone density assessment is critical for fracture risk prediction. Integrating the model with electronic health records (EHRs) could enable automated risk stratification, flagging patients with low HU values for further evaluation. Additionally, combining volumetric HU data with machine learning algorithms could predict long-term outcomes, such as the likelihood of vertebral fractures or the success of spinal fusion procedures. Such predictive capabilities could transform clinical decision-making, shifting the paradigm from reactive to proactive care in bone health management.

In summary, the deep-reasoning and learning-based model offers an efficient method for automated volumetric spinal bone density assessment. By achieving significant positive correlations with DXA-derived BMD, T-scores, and Z-scores, the model is scalable compared to traditional manual single-slice HU methods. With further validation, bone density data obtained from the model could be used in larger cohorts and inform surgical planning.

## Figures and Tables

**Table 1:** Average BMD and HU values across vertebral levels.

	BMD ( $\bar{x}$ ± SD)	vHU ( $\bar{x}$ ± SD)
**L1**	1.009 ± 0.196	140.823 ± 63.618
**L2**	1.062 ± 0.376	144.466 ± 51.281
**L3**	1.171 ± 0.223	150.221 ± 52.480
**L4**	1.209 ± 0.250	157.605 ± 53.263

**Table 2:** L1 correlation matrix.

	L1 vHU	L1 BMD	L1 T-Score	L1 Z-Score
**L1 vHU**	1	0.6633	0.6609	0.5168
**L1 BMD**	0.6633	1	0.9976	0.8063
**L1 T-score**	0.6609	0.9976	1	0.8216
**L1 Z-score**	0.5168	0.8063	0.8216	1

**Table 3:** L2 correlation matrix.

	L2 vHU	L2 BMD	L2 T-Score	L2 Z-Score
**L2 vHU**	1	0.7524	0.7549	0.5969
**L2 BMD**	0.7524	1	0.9389	0.8628
**L2 T-score**	0.7549	0.9389	1	0.8249
**L2 Z-score**	0.5969	0.8628	0.8249	1

**Table 4:** L3 correlation matrix.

	L3 vHU	L3 BMD	L3 T-Score	L3 Z-Score
**L3 vHU**	1	0.613	0.6136	0.441
**L3 BMD**	0.613	1	0.9972	0.8465
**L3 T-score**	0.6136	0.9972	1	0.8577
**L3 Z-score**	0.441	0.8465	0.8577	1

**Table 5:** L4 correlation matrix.

	L4 vHU	L4 BMD	L4 T-Score	L4 Z-Score
**L4 vHU**	1	0.6954	0.6942	0.5747
**L4 BMD**	0.6954	1	0.985	0.8676
**L4 T-score**	0.6942	0.985	1	0.8899
**L4 Z-score**	0.5747	0.8676	0.8899	1

## Data Availability

All data and materials support their published claims and comply with field standards and are available for review.
